# Preventing sight-threatening ROP: a neonatologist's perspective

**Published:** 2017

**Authors:** Ashok Deorari, Brian A Darlow

**Affiliations:** Professor: Department of Paediatrics, WHO Collaborating Centre for Training and Research in Newborn Care), All India Institute Of Medical Sciences, New Delhi, India.; Emeritus Professor of Paediatrics: University of Otago, Christchurch, New Zealand.

**Figure:**
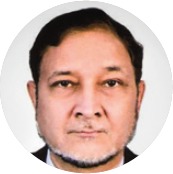
Ashok Deorari

**Figure:**
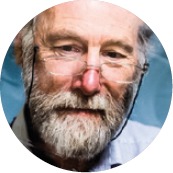
Brian A Darlow

**Neonatal care during the first hours and weeks of life determines a preterm baby's chances of avoiding retinopathy of prematurity and its complications. Oxygen management and low-cost interventions make all the difference.**

**Figure:**
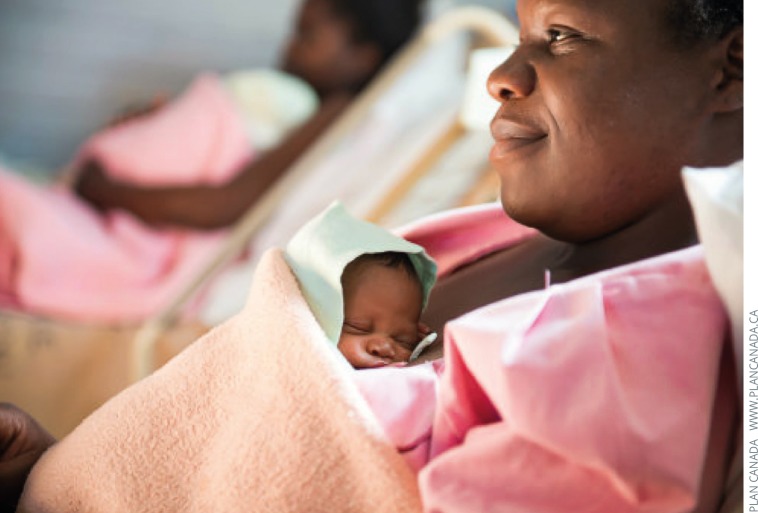
Supportive care practices, such as kangaroo care, are central to the management of infants who are at risk of ROP.

It is recognised that the number of new cases (incidence) of retinopathy of prematurity (ROP) varies considerably between different intensive care neonatal units, even those with similar characteristics in terms of the equipment and clinical staff available. Whilst there may be several other reasons for this, one reason we can be certain about is that there are differences in newborn care practices between units. Routinely implementing standard interventions that are known to prevent ROP will improve outcomes.

## Preventing ROP before delivery

A course of steroids, given to mothers likely to give birth prematurely, improves survival and reduces the complications of prematurity, including ROP. Antenatal steroids should be routine for mothers likely to give birth to a baby of less than 35 weeks' gestation.

**Figure:**
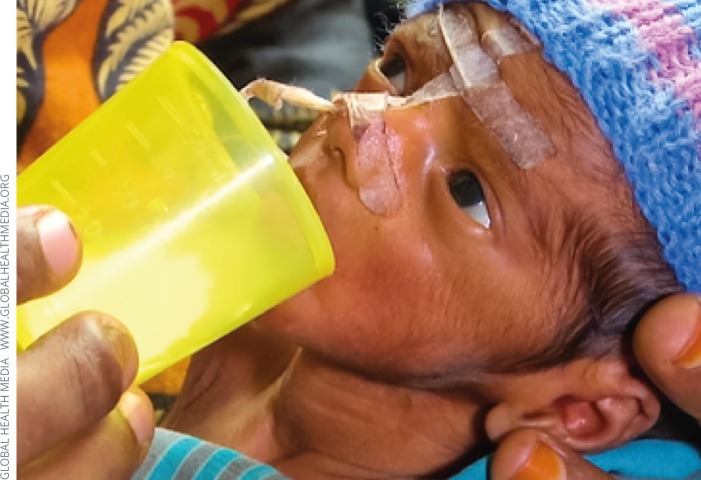
If preterm babies cannot be breastfed, they can be given small amounts of breast milk by cup.

## Risk factors for ROP

In addition to ROP, preterm babies can have other serious complications, including changes in the brain, chronic lung disease, and severe infection of the gut. Interventions and better care practices which aim to prevent one problem, for example infection, frequently also reduce the incidence of another, such as ROP.

The main risk factor for ROP is prematurity, but this is difficult to prevent. However, other factors such as exposure to too much oxygen, infection, and poor weight gain after birth also increase the risk. Controlling these factors requires high quality neonatal care, which can be summarised as POINTS of Care:

- **P**ain control
- **O**xygen management
- **I**nfection control
- **N**utrition
- **T**emperature control
- **S**upportive care

Before describing how these risk factors can be controlled during a baby's stay in the neonatal unit, it is important to understand the following:

- How to deliver and monitor oxygen levels in the blood
- How to prevent ROP immediately after preterm birth.

## Delivering and monitoring oxygen levels

**Figure 1:**
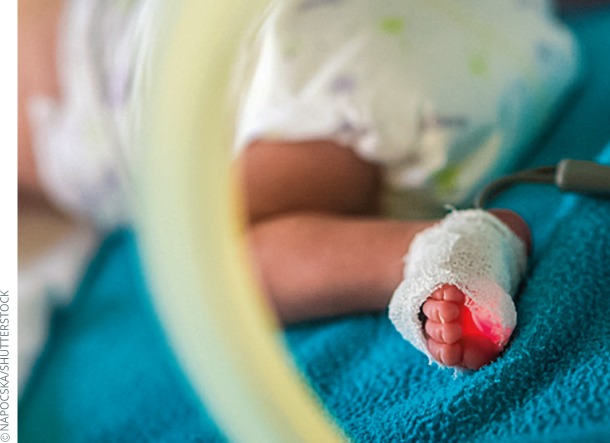
A pulse oximeter is attached to a preterm baby's foot

**Figure 2:**
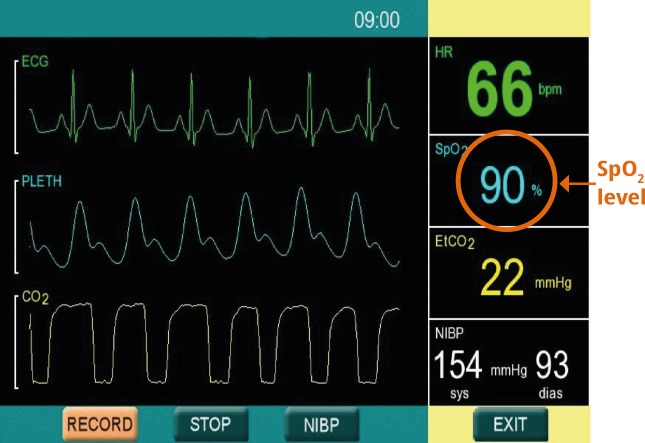
The oxygen saturation is expressed as a percentage (90%)

Oxygen saturation (SpO_2_) is a measurement of the proportion of haemoglobin in arterial blood that is carrying oxygen. The air we breathe is 21% oxygen and – in healthy adults – this is enough to ensure that all the haemoglobin in the arterial blood is carrying oxygen (i.e., an SpO_2_ of 100%). SpO_2_ can be measured at any age using a pulse oximeter. For preterm babies, the probe is usually attached to the foot (Figure 1). The SpO_2_ level is shown on a display monitor (Figure 2).

In the womb, a baby's SpO_2_ is less than 100%; it is usually around 50–70%. This is entirely normal. After birth, the SpO_2_ in a healthy baby increases gradually to around 100% at 10 minutes.

If the saturation is lower than it should be at any time during neonatal care, additional oxygen can be given at varying concentrations. This is called supplemental oxygen. In preterm babies, an SpO_2_ of 95–100% can damage developing blood vessels in the retina, leading to ROP, and can damage the lungs and brain. A low SpO_2_ can also lead to brain damage. Careful administration and oxygen monitoring from immediately after birth are therefore essential in preterm babies. Alarms on the monitor should be set so that they sound if the SpO_2_ levels are too high (95% or above) or too low (88% or less). This alerts the neonatal team so that they can address the problem as quickly as possible.

## Preventing ROP during the first hour after preterm birth

The first hour of life has been called the ‘golden hour’ because several low-cost interventions greatly improve outcomes (Table 1). These include delayed clamping of the umbilical cord, keeping babies warm, and gentle respiratory support. Protocols are essential so that staff can work as a co-ordinated team. Routine resuscitation of term and moderate-to-late preterm babies begins with gentle ventilation with a bag and mask, using air.

Preterm infants <32 weeks should receive ventilation with a bag and mask and 30% oxygen, modifying the concentration of oxygen given to meet time-specific oxygen saturation targets (Table 2). Giving 100% oxygen is not necessary for most preterm babies. Ideally, there should be equipment to mix air and oxygen (blenders) in the delivery room. If the baby is not breathing well, or the heart rate is dropping, the concentration of oxygen given can be increased to 100% and then reduced as soon as possible.

## Preventing ROP in the neonatal unit: POINTS of Care

There are a number of low-cost, effective practices that can reduce the risk of ROP. Many of these ‘POINTS of Care’ (see below and in Table 3, overleaf) help to keep babies stable and reduce wide fluctuations in blood oxygen levels so that extra oxygen is not needed.

**Pain** makes babies unstable. It can increase the need for oxygen and worsen respiratory distress. See Table 3.

**Oxygen.** The World Health Organization recommends that for preterm babies with a gestational age of less than 32 weeks, the SpO_2_ should be not be lower than 89% and not higher than 94% (the upper limit is 94% to prevent ROP). This means that the alarms on the monitors should be set at 88% and 95% so that they will sound if the oxygen saturation goes below or above this recommended range. Pulse oximeters are easy to use. They should be used for all preterm infants receiving supplemental oxygen. If there is not enough equipment to monitor oxygen levels in all babies, priority should be given to those who are unwell, those being handled, and those being given higher concentrations of supplemental oxygen.

**Table 1:** Labour ward and delivery room interventions

Intervention	Explanation
**Antenatal corticosteroids** for preterm births (< 35 weeks' gestation)	Reduces mortality, the severity of respiratory distress and other complications
**Delay clamping the umbilical cord** by 30–60 seconds in vigorous preterm infants	Decreases some complications (IVH, NEC) and reduces the need for blood transfusion
**Keep preterm babies warm.** Use a plastic bag or occlusive wrapping (Figure 2, p. 54)	Maintaining normal temperature (36.5–37.2°C) reduces the risk of severe ROP and other complication
**Gentle respiratory management**	This avoids injury to the lungs. Most newborns are not pink at birth. If they are breathing well, the colour will improve in 5–10 minutes

**Table 2:** Target oxygen saturation levels (SpO_2_) in preterm infants during the first 10 minutes after birth

Time after birth	Oxygen saturation* (range)
2 min	55–75%
3 min	65–80%
4 min	70–85%
5 min	80–90%
10 min	85–95%
*The proportion of haemoglobin in arterial blood that is carrying oxygen	

**Table 3:** Neonatal care best practices

Intervention	Explanation
**P**ain: Avoid and prevent painful episodes	Reduce unnecessary painful procedures. Anticipate pain and prevent it by swaddling and use of oral sucrose or glucose
**O**xygen management	Ensure that the oxygen saturation is between 89% and 94%
**I**nfection control	Apply infection control procedures, including hand washing by all
**N**utrition: Improved nutrition with breast milk	Use mothers' own breast milk but provide extra protein and calories
**T**emperature control	Keep the baby warm from immediately after birth, by wrapping, using a hat and keeping the baby in an incubator, or under a warmer
**S**upportive care	Includes good positioning of the baby in an incubator or cot and the use of kangaroo care
Other: Minimise blood transfusions	Reduce blood sampling and the volume of blood taken. Blood transfusions have been linked with ROP

**Infection** can be reduced by hand washing (or alcohol rubs after an initial wash) on entering the NICU and before and after handling each baby. This must be practiced by all. Measures to reduce skin breakdown, sterile techniques for intravenous lines, and careful use of antibiotics all reduce infection. Having an infection control team, headed by a senior nurse, is often beneficial.

**“Making sure preterm babies receive high quality care requires experienced nurses who do not have to look after too many babies.”**

**Nutrition.** Good nutrition and growth are essential for short- and long-term outcomes. There are many benefits of feeding preterm babies their own mother's breast milk, including lower rates of ROP. For babies below 1,000 g intravenous feeding may also be required.

**Temperature control.** Both high and low temperatures make babies unstable and can increase the need for oxygen. It can also worsen respiratory distress.

**Supportive care** practices are those which keep babies comfortable and stable, including kangaroo care and ensuring that babies' limbs are supported (pp. 53–54).

## General aspects

In high-income countries, changes in how services for preterm infants are organised have improved the survival of preterm babies and reduced complications, including severe ROP. These include developing centres of excellence for the sickest preterm babies and better care of babies while they are being transported to or between neonatal units.

Providing better neonatal care requires team work between different health professionals (doctors, nurses, allied health workers) and working closely with parents and health authorities.

All units should have agreed protocols for important aspects of newborn care. These should be based on the best evidence available, i.e., from high quality clinical trials and systematic reviews. Good data collection methods are also needed in order to monitor trends and compare outcomes with similar neonatal units. Sharing information and best practices is easier if several units establish formal networks.

Making sure that preterm babies receive high quality care requires experienced nurses who do not have to look after too many babies. Ideally, one experienced neonatal nurse should not look after more than two sick infants. Working with parents is also very important (pp 60–61). There are many neonatal practices which can reduce the risk of severe ROP and so prevent blindness.
